# Functional characteristics and microbiological viability of foam‐mat dried Bambara groundnut (*Vigna subterranea*) yogurt from reconstituted Bambara groundnut milk powder

**DOI:** 10.1002/fsn3.951

**Published:** 2019-09-30

**Authors:** Zolelwa Hardy, Victoria A. Jideani

**Affiliations:** ^1^ Department of Food Science and Technology Cape Peninsula University of Technology Bellville 7535 South Africa

**Keywords:** Bambara groundnut, foam‐mat drying, functionality, Gum Arabic, methylcellulose, yogurt powder

## Abstract

The functional, nutritional, and physical characteristics of foam‐mat dried Bambara groundnut (*Vigna subterranea*) yogurt were investigated. Bambara groundnut powdered yogurt (BGNPY) was produced using Bambara groundnut milk powder (BGNMP) and Bambara groundnut milk (BGNM). BGNMP was reconstituted with water (1:5). The reconstituted BGN milk (BGNM‐R) and original nonreconstituted BGNM (BGNM‐NR) were inoculated with normal yogurt culture while held at a temperature of 45°C and incubated for 24 hr at 35°C. The BGN yogurts were dried employing the foam‐mat drying process with gum arabic (6%) and methylcellulose (0.5%) as foaming agents and dried at 50°C for 24 hr. The BGN powdered yogurt from reconstituted milk (BGNPY‐RM) and BGN powdered yogurt from nonreconstituted milk (BGNPY‐NRM) were evaluated for functional, nutritional, thermal, and physical characteristics. Water absorption (1.27 and 1.31 g/g) and water solubility (73.3. and 71.22 100/g) index of the powdered yogurts did not differ significantly, while a significant (*p* < 0.05) difference was observed for the Tg of BGNPY‐R and BGNPY‐NR. Nutrient composition of BGNPY‐R and BGNPY‐NR had no significant (*p* > 0.05) difference, while ash differed significantly (*p* < 0.05). Particle size and particle size distribution of BGNPY‐R and BGNPY‐NR had no significant (*p* > 0.05) difference. Probiotic viability of BGNPY‐R (7.2 log cfu/ml) remained above the minimum recommended dosage (6 log cfu/ml).

## Graphical abstract

The study investigates the functionality of Foam‐mat powdered yogurt produced from reconstituted BGNMP in comparison with that produced from liquid BGN milk. BGNPY‐R and BGNPY‐NR showed favorable WAI, WSI, and wettability necessary for reconstitution. Only BGNPY‐R could be deemed as a probiotic containing food, as the probiotic viability was well above the minimum recommended dosage.

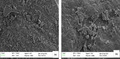

## 1 INTRODUCTION

Yogurt is a milk product produced from fermentation of milk sugars into lactic acid by the addition of yogurt starter culture containing *Streptococcus thermophilus* and *Lactobacillus delbrueckii* ssp. *Bulgaricus* (Körzendörfer, Nöbel, & Hinrichs, 2017; Mckinley, 2005). Other lactic acid bacteria include less traditional microorganisms, such as *Lactobacillus helveticus* and *Lactobacillus delbrueckii* ssp *Lactis,* mixed with the starter culture, which is used in some other countries (Mckinley, 2005). Cultured milk products such as yogurt were initially manufactured as a means of preserving the nutrients in milk. However, it was soon discovered that fermenting with different microorganisms allows the development of a wide range of products with different flavors, textures, consistencies, and, more recently, health attributes (Tamime, 2002). Yogurt starter culture is reported to have antagonistic behavior toward undesirable microorganisms found in the intestines due to some metabolites produced during fermentation (Kumar & Mishra, 2004a, 2004b; Ng, Yeung, & Tong, 2011). Yoghurts come in a variety of textures (e.g., liquid, set, and smooth), fat contents (e.g., luxury, low‐fat, virtually fat‐free), and flavors (e.g., natural, fruit, cereal). This versatility, together with their acceptance as a healthy and nutritious food, has led to their widespread popularity across all population subgroups (Mckinley, 2005).

The versatility of the existence of yogurt has also encouraged and promoted production of yogurt originating from plant sources (milk legume) as a substitute to the animal milk. There is an inadequate supply and shortage of food protein in Africa and the other developing countries, mainly because animal protein including milk, meat, and eggs is high‐priced and relatively difficult to acquire (Aremu et al., 2010). There has been a constant search for unconventional legumes such as Bambara groundnut legume as a new protein source and almost nutritionally balanced food material for their use as a functional food ingredients and nutritional supplement (Yao et al., 2015).

Bambara groundnut (BGN) is one of the underutilized legume seeds, which makes a complete food as it contains an important source of all nutrients classes, such as protein (18–24%), oils (6%–8.5%), carbohydrates (53%–69%), and minerals (2.5%–3.5%) [Ogundele, Minnaar, & Emmambux, 2017; Gulzar & Minnaar, 2017]. The legume crop is botanically known as *Vigna subterranea (L.) Verdc*., a member of the Fabaceae family, originating from West Africa (Yao et al., 2015). The legume is climate smart, as it is drought tolerant and grows under adverse climatic and soil conditions (Gulzar & Minnaar, 2017). It is a rich source of protein that could help alleviate nutritional problems in these areas (Yao et al., 2015). The seeds can be used to produce vegetable milk that is comparable to other vegetable milks already in the industry such as soya milk with respect to sensory attributes (color and taste) (Okudu & Iloh, 2016), which then can be used to produce legume‐based yogurt (Massawe, Mwale, Azam‐Ali, & Roberts, 2005). Brough, Azam‐Ali & Taylor (1993) reported milk prepared from BGN to be of most preference in comparison with milk produced from cowpea, pigeon pea, and soybean with respect to taste and color. Furthermore, Agunbiade, Amosu, Degun, and Omeonu (2011) reported BGN milk to be acceptable to consumers and recommended as weaning food. Quasem, Mazahreh, and Abu‐Alruz (2009) further stated that major legumes that have been used to produce vegetable milk include cowpeas, winged bean, soybeans, groundnuts, and melon seeds. Murevanhema and Jideani (2014) studied the shelf life stability of BGN milk and reported fermented BGN milk with lactic acid as a probiotic beverage (Murevanhema and Jideani (2012). However, being a liquid, the shelf life could be reduced due to high moisture content. As a form of water reduction, foam‐mat drying technology will be employed for this study. Foam‐mat drying is a dehydration process of converting liquid or semi‐fluid foods into dried fine powder. This is accomplished through the addition of stabilizing and foaming agent, in turn also incorporating air into the food system. The food then becomes dry by introducing the heated air, resulting into a dry flake that can be further milled into a fine powder (Lobo et al., 2017; Ng & Sulaiman, 2018). The drying rate is dependent on the foam porosity and foam density, which is directly affected by the concentration of dissolved solids in a food system (Dehghannya, Pourahmad, Ghanbarzadeh, & Ghaffari, 2018). This process encourages an increase in the rate of heat transfer at lower temperature and final powder produced is capable of instant rehydration using cold water, due to increased porous structure. Nutrients are retained and excessive, unwanted browning on specific foods can be avoided as a result of decreased drying time. Moreover, foam‐mat drying technology is suitable for heat sensitive foods and more interest is developing around this technology due to the straightforward processing technique it provides as well as its cost‐effectiveness (Abbasi & Azizipour, 2016).

The need for dried yogurt is gradually increasing, not only for an increased shelf life, but also to provide convenience during packaging, handling, and transportation compared to liquid yogurt because the weight and volume of the product are less in its dried form. However, during the drying process the viability of the yogurt microflora on the final powder requires critical monitoring, highly influenced by the drying temperatures. The authors Piaia, Antoine, Mateos‐guardia, Leplingard, and Lenoir‐wijnkoop (2003) also reported that heat treatment temperatures of 60, 65, and 75°C for a period of 20 min after fermentation reduce lactic acid bacterial cells by 73%, 99%, and 99.99%, respectively. Currently, there is not much research published on foam‐mat dried yogurt and specifically from BGN milk powder (BGNMP); therefore, the primary objective of this study was to investigate the functional and microbiological viability of foam‐mat dried BGN powdered yogurt produced from reconstituted Bambara groundnut milk powder (BGNMP‐R) and from nonreconstituted Bambara groundnut milk (BGNM‐NR), which was used as the control.

## 2 MATERIALS AND METHODS

### 2.1 Source of materials and equipment

Methylcellulose and gum arabic from acacia tree were purchased from Sigma, South Africa. BGN seeds were purchased from Trio trade, Johannesburg, South Africa. Chemicals were purchased at CJP Chemicals, DuPoint, Tate & Lyle, South Africa. The yogurt starter culture was purchased from Danisco, Cape Town. Equipment used for the experiments was obtained from the Food Technology Department of Cape Peninsula University of Technology, South Africa.

### 2.2 Production of Bambara groundnut milk

Bambara groundnut milk was produced according to the in‐house patent method by Murevanhema and Jideani (2014). The BGN flour was rehydrated with normal cold tap water. The solution was blended using the Hallade SB‐4 111262 warring blender and allowed to stand at room temperature (21 ± 2°C) for 2 hr. The resultant slurry was filtered using fine cheese cloths. The filtrate was allowed to stand for 10 min to allow the particles to settle. The supernatant was then homogenized using a Cadmach Ahmedabad‐45 CMCM5 colloid mill. The solution was monitored for pH and total solids (TS), using the Crison, GLP 21 pH meter, Barcelona, and a refractometer (Bellingham Stanley eclipse 45–06). The solution was further mixed with ingredients and boiled at 100°C for 20 min on a hot plate stove, while stirring. The boiled solution was referred to as BGNM. The milk was instantly transferred into sterile glass bottles and immediately cooled to 4–8°C. The BGN milk samples were stored under refrigeration temperature (4–8°C) and were further monitored for TS and pH. The TS and pH of BGN milk were evaluated for the purpose of spray drying. The data acquired did not further undergo statistical analysis.

### 2.3 Spray drying of the Bambara groundnut milk

Bambara groundnut milk powder (BGNMP) was produced using method by Hardy & Jideani (2017). The TS of BGN milk as described in Section 2.2 was adjusted to 10% using corn maltodextrin (20 dextrose equivalent) as a drying carrier. BGN concentrated milk was preheated (60–80°C) and homogenized (85 rpm) for 20 min using a water bath. The concentrated BGNM was dried employing the spray drying technology, and the final dried BGNMP particles were collected from the cyclone and chamber and stored at room temperature (21 ± 2°C) until further utilization.

### 2.4 Reconstitution of Bambara groundnut milk powder

Previously prepared BGNMP (Section 2.3) was reconstituted (water with BGNMP) following the food component material balance equation (Equation (1)) method reported by Saravacos and Maroulis (2011). The moisture content of BGNMP before and after drying was measured according to AOAC (2005) method (934.01). The identified water (moisture) loss after drying was then used to establish the reconstitution parameters (water and BGNMP).

(1) (Total component in)-(Total component out)=Total component accumulated

where “total components in” represents initial product before processing (BGNM), “total component out” represents lost water through processing, and total component accumulated represents final moisture content of BGNMP. Consequently, BGNMP was added to water at a ratio of 1:5 (w/v).

### 2.5 Stability characterization of Bambara groundnut milk powder using Turbiscan

The stability of reconstituted BGNMP was analyzed using a Turbiscan Vertical Scan M.A 2000. Reconstituted (1:5 w/v) BGNMP (7 ml) was transferred into a Turbiscan glass sample tube (zone 20–40 mm) and closed tightly with a cap. The tube was inserted inside the cell and the detection head analyzer scanned the entire height of tube containing sample, acquiring transmission and backscattering data every 40 μm at 5 min intervals for 40 min at 20°C (Blijdenstein, Hendriks, van der Linden, van Vliet, & van Aken, 2003; Blijdenstein, Zoet, van Vliet, van der Linden, & van Aken, 2004). The destabilization was determined by means of scanning analyzing detection head, for the identification of coalescence or flocculation (due to particle size variation), as well as sedimentation and creaming (due to particle migration). The detection head passes light through the sample, which is received by the transmission detector, while the backscattering detector measures the light scattered backward by the sample. Measurements were done in duplicate.

### 2.6 Fermentation of BGN milk and Reconstituted BGN milk powder into BGN yogurt

Bambara groundnut milk and reconstituted (1:5 w/v) BGNMP (400 ml) in 500 ml schott bottles were warmed to 45°C in a water bath for 15–20 min. The BGNMP‐R and BGNM‐NR were aseptically inoculated with yogurt microflora and incubated at 35°C in a water bath for 24 hr. The fermented BGNM‐R and BGNM‐NR were immediately cooled in ice and stored under refrigeration at 3–4°C (Murevanhema & Jideani, 2012).

### 2.7 Drying of BGN yogurt into powder employing foam‐mat drying process

Bambara groundnut powdered yoghurts (BGNPY) were produced using the Krasaekoopt and Bhatia (2012) method with some modification. Foam‐mat drying technique was performed employing the optimized formulation. Gum arabic (6%) and methylcellulose (0.5%) were used as foaming agents. Fermented BGNM‐R and BGNM‐NR (Section 2.6) were mixed with the foaming agents, blended using a Silverson Model L4R Homogenizer for 10 min, and transferred into Teflon metal trays. The trays containing the foamed mixture were dried (Cabinet dryer, Model 1069616) at 50°C for 24 hr. The dried yogurt flakes were ground into fine powder using a Royals Worcester mortar and pestle. The fine BGN powdered yogurt was placed in sealed zip lock bags and stored till further analysis. The dried BGN yogurt was referred to as Bambara groundnut powdered yogurt (BGNPY). BGNPY from reconstituted BGN milk powder was referred to as (BGNPY‐R), while BGNPY from nonreconstituted BGN milk was referred to as (BGNPY‐NR).

### 2.8 Proximate analysis of Bambara groundnut powdered yoghurts

Protein determination was done using Kjeldahl method 920.53 (N X 6.25) as described in AOAC (1992), total dietary fiber according to AOAC (1997) method 985.29, total sugar according to AOAC (2003) method 982.14, and moisture, ash, and total fats according to AOAC (2005) method 934.01, 923.03, and 996.06, respectively. The carbohydrate was determined by difference and energy was determined according to the method of Edgah, Kirk, Sawyer, and Pearson (1981).

### 2.9 Functional characterization of Bambara groundnut powdered yoghurts

#### 2.9.1 Water absorption index (WAI) and water solubility index (WSI)

Water absorption index and WSI of the BGN powdered yogurt from reconstituted milk (BGNPY‐R) and BGN powdered yogurt from non‐reconstituted milk (BGNPY‐NR) were determined using the method of Kaushal, Kumar, and Sharma (2012). BGNPY‐R and BGNPY‐NR (2.5 g) were dispersed in 30 ml of distilled water, using a glass rod, and cooked at 90°C for 15 min in a heating metal. The cooked paste was cooled to room temperature and transferred to tared centrifuge tubes, and then centrifuged at 3,000 *g* for 10 min. The supernatant was decanted for the determination of its solid content into a tared evaporating crucible dish and the sediment was weighed. The weight of the dry solids was recovered by evaporating the supernatant overnight at 110°C. WAI and WSI were calculated using Equations (2) and (3).

(2) $$\text{WAI}\left(\text{g}/\text{g}\right)=\frac{\text{Weight of sediment}}{\text{Weight of flour sample}}$$

(3) $$\text{WSI}\left(\text{g}/\text{g}\right)=\frac{\text{Weight of dissolved solids in supernatant}}{\text{Weight of flour}}$$

#### 2.9.2 Wettability

Wettability was determined using the procedure described by Jinapong, Suphantharika, and Tamnong (2008). Distilled water (100 ml) at a temperature of 25 ± 1°C was poured into a 250 ml glass beaker. A glass funnel held on a retort stand was set over the beaker with the height between the bottom of the funnel and the water surface of 10 cm. A test tube was placed inside the funnel to block the lower opening of the funnel. BGNPY‐R and BGNPY‐NR weighing 0.1 g were placed around the test tube and the tube was lifted, while the stop watch was started at the same time. Time taken for the powder to become completely wet, and for all the powder particles to penetrate the surface of the water, was recorded.

#### 2.9.3 Bulk density measurement of BGN powdered yogurt

BGNPY‐R and BGNPY‐NR each weighing 2 g were placed into graduated syringes, and sufficient pressure was applied to pack the content in the syringes. The final volume of the sample in each syringe was recorded, and bulk density was expressed as grams per milliliter (g/ml) (Parrott & Thrall, 1978).

### 2.10 Glass transition temperature of BGN powdered yogurt

The glass transition temperature (Tg) was determined using the method described by Shrestha, Howes, Adhikari, and Bhandari (2007) with some modifications. A Perkin Elmer Pyris 1 Differential Scanning Calorimeter (DSC) 6000 equipped with Intracooler 2P was used to evaluate the glass transition temperature of BGNPY‐R and BGNPY‐NR samples. The purge gas used was dry nitrogen (20 ml/min). Heat flow verification was performed using indium melting point (156.60°C) and zinc melting point (419.47°C) standards at heat scan rate of 10°C/min. About 10 mg of BGNPY‐R and BGNPY‐NR was weighed into a 50 μl DSC aluminum pan and press sealed with a lid using a DSC sample press. The thermal scanning of the equilibrated samples was carried out alongside a sealed empty pan used as a reference. The thermal scanning of BGN powdered yogurt samples commenced from ‐20°C to 200°C at 10°C/ in triplicates and the onset, endset values were calculated in the DSC thermo‐gram, and the Tg value was determined as half ∆Cp method at half the extrapolated change in specific heat using the Pyris software.

### 2.11 Physical characterization of Bambara groundnut powdered yogurt

#### 2.11.1 Particle size measurement of BGN powdered yogurt

The particle size and particle size distribution were analyzed using a Leo 1430VP scanning electron microscope (SEM) at Stellenbosch University, South Africa. Prior to imaging, the samples were mounted on a stub with double‐sided carbon tape. The samples were then coated with a thin layer of gold in order to make the sample surface electrically conducting. Beam conditions during surface analysis were 7 kV and approximately 1.5 nA, with a spot size of 150.

Particle sizes and particle size distribution of BGNPY‐R and BGNPY‐NR were evaluated using images obtained from the SEM. The diameters of the particle sizes were measured individually according to the method of Tcholakova, Denkov, and Danner (2004). BGNPY particle sizes were obtained in terms of volume‐surface mean diameter (*d*_3_,_2_) (Equation (4)) and equivalent volume mean diameter (*d*_4_,_3_) (Equation (5)).

(4) $$d_{3,2}=\frac{\sum n_{i}{\text{d}}_{i3}}{\sum n_{i}d_{i2}}$$

(5) $$d_{4,3}=\frac{\sum n_{i}d_{i4}}{\sum n_{i}d_{i3}}$$

#### 2.11.2 Water activity evaluation of BGNPY‐RM and BGNPY‐NRM

The water activity (*a*_w_) of BGNPY‐R and BGNPY‐NR was measured using the Novasina Ms 1 Set *A*_w_ meter, which uses a cell protection filter to measure *a*_w_. Salt humidity standards of 53%, 75%, and 90% relative humidity were used to calibrate the measurement cell. BGNPY‐R and BGNPY‐NR (5 g) were transferred into a sample dish and placed inside the Novasina analyzer, and the cell measuring protection filter was immediately closed. The *a*_w_ reading was observed after period of 60–80 s. The test was performed in triplicate (Novasina General Catalogue, 2012).

### 2.12 Microbiological Measurements of Bacterial Load in BGN Powdered yogurt

The pour plate method was employed for the enumeration of lactic acid bacteria in fermented BGNM‐R and BGNM‐NR before drying, as well as in BGNPY‐R and BGNPY‐NR after drying. BGNPY‐R and BGNPY‐NR (10 g) were weighed into 90 ml sterile ringers' solution (10^−1^) and mixed well. Then, a series of dilutions (10^−2^ to 10^−8^) were prepared by transferring 1 ml aliquot from the 100 ml sample solution into 9 ml sterile ringers' solution as the second dilution (10^−2^), until last dilution (10^−8^). For each dilution, a 1 ml aliquot was carefully and aseptically transferred into the base of a labeled sterile Petri dish. Then separately, approximately 15 ml of precooled De Man Rogosa Sharpe (MRS) was poured for the enumeration of mesophilic counts and carefully swirled to mix well. Once all plates were allowed to solidify, they were incubated in an inverted position, at 30°C for 5 days in an aerobic atmosphere supplemented with carbon dioxide (Baylis, 2007). Counting of all typical colonies was performed using BOECO CC1, BOE 515700, Germany and colony counter, while a control was carried out for each analysis. All measurements were performed in triplicate. Only plates containing colonies from 25 to 250 were counted, a standard countable range for enumerating colony forming units on an agar plate using the pour plate technique without introducing error.

### 2.13 Data analysis

All measurements were carried out in triplicate, and results were expressed as mean ± *SD*. Multivariate analysis of variance (MANOVA) was employed to determine mean differences among treatments. Duncan's multiple range test was used to separate means where differences existed (IBM Corp 23, 2013).

## 3 RESULTS AND DISCUSSION

### 3.1 Stability of reconstituted Bambara groundnut milk

**Figure 1:**
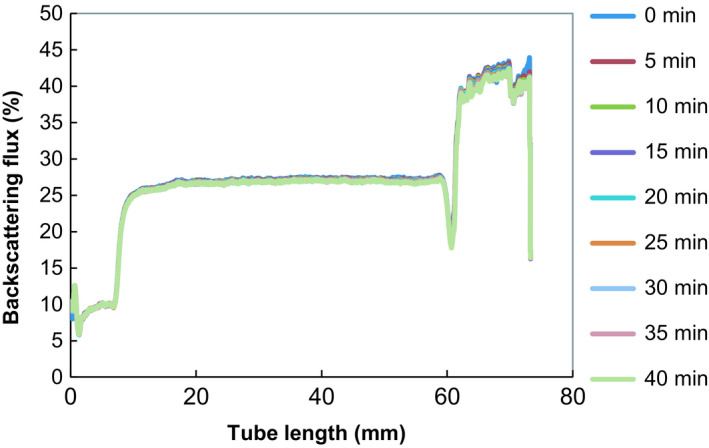
Turbiscan backscattering multiple stability scan profile of reconstituted Bambara groundnut milk powder

Figure 1 is the Turbiscan profile of reconstituted BGNM indicating the stability curves as backscattering (BS) flux percentage (%). The BS% of the reconstituted milk was approximately 25.6% presented on the *y*‐axis, which also represented the solids in reconstituted BGNM solution. The reconstituted milk was quite stable observed on the *x*‐axis (10–60 mm), indicated by the straight curve of multiple lines overlaying on top of each other. This implies that the particles were evenly dispersed in the solution.

Stability is studied to evaluate the homogeneity of an emulsion or solution, which further identifies creaming, sedimentation, coalescence, and the flocculation mechanism occurring in that solution (McClements, 2004). The creaming instability can be identified by concentration of particles migrating to the top of the solution, where as in sedimentation particles settle to the bottom of the solution. Flocculation is the aggregation of particles and coalescence involves merging of two particles into one large particle (McClements, 2004; Robins, Watson, & Wilde, 2002). Liang et al. (2014) reported that the creaming and sedimentation mechanism is said to occur due to the gravitational and or centrifugal force separation that out weights the diffusion of droplets or particles.

The stability of reconstituted BGN milk can then be attributed to what was reported by Klinkesorn, Sophanodora, Chinachoti, and McClements (2004) that texture modifiers used as ingredients increased the viscosity of the continuous phase emulsion, thereby slowing down gravitational separation of droplets. It was further stated that the stabilizing action of maltodextrin is by viscosity modification or gelation of an aqueous continuous phase, which explains the stability of the reconstituted BGN milk, as maltodextrin was one of the main ingredients.

### 3.2 Proximate composition of Bambara groundnut powdered yogurt

The chemical composition of BGNPY‐R and BGNPY‐NR is displayed in Table 1. The moisture, ash, total fat, monounsaturated fats, polyunsaturated fats, saturated fats, protein, total dietary fiber, total sugars, carbohydrates, and energy of BGNPY‐NR: BGNPY‐R are 9.1; 9.0%, 2.3; 2.5%, 1.4; 1.6%, 0.3; 0.4%, 0.6%; 0.6%, 0.5; 0.6%, 5.7; 4.8%, 4.2; 3.9%, 2.1; 2.2%, 75.3; 76.0% and 1,430; 1,433 kJ, respectively. The chemical composition of the BGNPY‐R and BGNPY‐NR was not significantly (*p* > 0.05) different, except for their ash content (*p* < 0.05). BGNPY‐R had higher ash content (2.52%), compared to BGNPY‐NR (2.29%). On the other hand, BGNPY‐NR had a noticeably higher moisture (9.1%), protein (5.7%), and total dietary fiber (4.2%) content compared to BGNPY‐R, which was not significant. The total dietary fiber content of BGNPY‐R (3.9%) and BGNPY‐NR (4.2%) was observed to be fairly high.

**Table 1:** Chemical composition (g/100 g product) of Bambara groundnut powdered yogurt from reconstituted and non‐reconstituted Bambara groundnut milk

	Proximate (%)	
Nutrient	BGNPY‐NR	BGNPY‐R
Moisture	9.1 ± 0.03^a^	9.0 ± 0.01^a^
Ash	2.3 ± 0.01^a^	2.5 ± 0.01^b^
Total fat	1.4 ± 0.14^a^	1.6 ± 0.13^a^
Monounsaturated fats	0.3 ± 0.04^a^	0.4 ± 0.02^a^
Polyunsaturated fats	0.6 ± 0.06^a^	0.6 ± 0.03^a^
Saturated fat	0.5 ± 0.04^a^	0.6 ± 0.08^a^
Protein	5.7 ± 0.20^a^	4.8 ± 0.28^a^
Total dietary fiber	4.2 ± 0.13^a^	3.9 ± 0.08^a^
Carbohydrates	75.3 ± 0.58^a^	76.0 ± 0.29^a^
Of which total sugars	2.1 ± 0.05^a^	2.2 ± 0.20^a^
Energy kJ	1,430. ± 1.42^a^	1,433 ± 4.94^a^

Note

Mean ± *SD* of triplicate determinations, mean values in the same row followed by different letters are significantly different (*p* < 0.05); BGNPY‐NR: Bambara groundnut.

This may be attributed to the foaming agents used, gum arabic, and methylcellulose. Soluble fibers, which are known for their good solubility in water, have been reported to include mucilage and gums (botanical, animal, and microbial). Gum arabic and methylcellulose are classified as botanical gums (Dhingra, Michael, Rajput, & Patil, 2011; Tosha & Yada, 2010), which were incorporated in the manufacturing of BGNPY‐R and BGNPY‐NR. Furthermore, methylcellulose is derived from highly purified forms of cellulose (Manthey & Xu, 2009), known to form part of insoluble dietary fiber (Dhingra et al., 2011). Therefore, the addition of these gums may have contributed to the high total dietary fiber. The fairly high protein of BGNPY‐R (4.8%) and BGNPY‐NR (5.7%) may also be associated with the gum arabic incorporated into the system. Williams and Phillips (2001) stated that gum arabic contains small amounts of protein, which is the important part of its structure.

### 3.3 The functional characteristics of BGNPY‐RM and BGNPY‐NRM

The WAI, WSI, wettability, and BD of BGNPY‐R and BGNPY‐NR were 1.27 g/g; 1.31 g/g, 73.30%; 71.22%, 15.70 s; 46.70 s and 0.87 g/ml; 0.82 g/ml, respectively. There was no significant (*p* < 0.05) difference on the WAI, WSI, and BD between the powdered yoghurts. However, a significant (*p* < 0.05) difference was observed in wettability between the two powdered yoghurts as shown in Table 2. BGNPY‐R was higher in wettability (15.70 s) compared to BGNPY‐NR (46.70 s). This may be linked to the statement reported by Hammes, Englert, Noreña, and Cardozo (2015), stating that the wetting of the surface of a solid food material by a liquid is assessed through contact angles and as is highly influenced by the surface composition of the food material. The authors further elucidated that hydrophobic component (e.g., lipids) at the surface produces high water contact angles, while hydrophilic components generate low contact angles with water. As observed, BGNPY‐R was reported to have higher fat (1.6%) content as compared to BGNPY‐NR (1.4%) (Section 3.2).

**Table 2:** Functional properties of BGNPY‐R and BGNPY‐NR

Powdered yogurt	WAI (g/g)	WSI (100/g)	Wett. (s)	BD (g/ml)
BGNPY‐R	1.27 ± 0.09^a^	73.30 ± 1.47^a^	15.70 ± 3.97^a^	0.87 ± 0.04^a^
BGNPY‐NR	1.31 ± 0.08^a^	71.22 ± 0.84^a^	46.70 ± 3.59^b^	0.82 ± 0.04^a^

Note

Mean values ± *SD* of triplicate determinations. Mean values in the same column followed by different letters are significantly (*p* < 0.05) different; BD: bulk density; BGNPY‐NR: Bambara groundnut powdered yogurt from fresh BGNM; BGNPY‐R: Bambara groundnut powdered yogurt from reconstituted BGNMP; WAI: water absorption index; Wett.: wettability; WSI: water solubility index.

Koç, Sakin‐Yılmazer, Kaymak‐Ertekin, and Balkır (2014) reported the functional properties of spray dried dairy yogurt powder, with wettability (374 s), solubility index of 68.7%. However, BGN powdered yoghurts showed much higher rehydration properties, with respect to WAI (1.27 g/g; 1.31 g/g), WSI (73.30%; 71.22%), and wettability (15.70 s; 46.70 s) for BGNPY‐R and BGNPY‐NR, respectively.

This may be linked to the gum arabic (6%) incorporated into the milk in production of BGN yogurt powders, reported to be readily soluble in water (Ward, Hanway, & Ward, 2005). Furthermore, it was also stated that methylcellulose is nonionic, an active molecule that has no electric charge (not affected by the hardness of water), but can also act as an emulsifier due to its hydrophilic as well as hydrophobic capabilities (Fernandez, Schebor, & Chirife, 2003). In addition, Koç et al. (2014) stated that the hydration properties of food parties can be influenced by the particle size material, a lower particle size results in an increase in the particle surface area causing high affinity with water, thus higher solubility. The particle size of BGNPY‐R and BGNPY‐NR was 105.46 and 114.86 μm, respectively (Section 3.5), and Koç et al. (2014) further reported the spray dried yogurt powder to contain a particle size of 93.053 μm, lower than that of BGNPY.

The BD of BGNPY‐RM (0.87 g/ml) and BGNPY‐NRM (0.82 g/ml) was higher compared to the BD of spray dried dairy yogurt (0.538 g/ml) reported by Koç et al. (2014). This may be associated with different processing techniques employed to obtain the yogurt powders, as BGN powdered yogurt was foam‐mat dried and further manually milled to a fine powder. The BD differences may also be attributed to the different yogurt compositions. Bulk density indicates load, a sample can carry, when allowed to rest directly on another (Kaushal et al., 2012), and high bulk density is a crucial obligation for commercial powders, beneficial in the cost reduction of packaging and shipping (Sharma, Jana & Chavan, 2012).

### 3.4 Glass transition temperature (Tg) characteristics of BGN powdered yogurt

**Figure 2:**
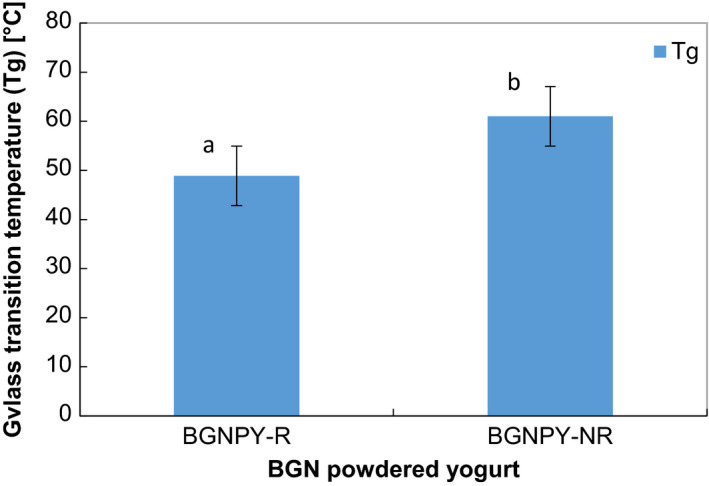
Glass transition temperature (Tg) of Bambara groundnut powdered yogurt (BGNPY)‐R and BGNPY‐NR

The glass transition temperature (Tg) of BGNPY‐R and BGNPY‐NR was 48.88°C and 61. 01°C, respectively, shown in Figure 2. There was a significant (*p* < 0.05) difference in the Tg of BGNPY‐R and BGNPY‐NR. BGNPY‐NR had the highest Tg of 61.01°C compared to BGNPY‐R (48.88°C), which implies that BGNPY‐NR is more heat stable than BGNPY‐R.

The stability of a milk powder is closely associated with its physical state, which includes physical changes such as sticking and caking resulting in collapsing of the structure. Deformed physical stability of milk powders adversely affects their quality and shelf life (Hogan, Famelart, O'Callaghan, & Schuck, 2010). The phenomenon of stickiness and caking occurs due to plasticization of milk powder surface particles by water, allowing inter‐particle binding and formation of clusters. When the powder is in its glassy stable state, it has high viscosity and the contact time between particles is prolonged. A drastic decrease in viscosity over the Tg range reduces the contact time between particles, causing inter‐particles to join resulting and stickiness and caking (Roos, 2001).

The Tg phenomena (glassy state to rubbery state) powders only occur when powders are exposed to temperatures above their Tg value (Foster, Bronlund, & Paterson, 2006). Therefore, both BGNPY‐R and BGNPY‐NR are less susceptible to physical deformation of caking and stickiness and can be deemed stable as their Tg is above room temperature, which is their recommended storage temperature, thus having the potential of an increased shelf life.

### 3.5 Physical characteristics of BGN powdered yogurt

In this study, the physical properties evaluated included particle size, water activity, and color parameters, of Bambara groundnut powdered yogurt. These attributes of BGN powdered yogurt are discussed in the following sections.

#### 3.5.1 Particle size characteristics of BGN powdered yogurt

The surface area mean diameter (*d*_3,2_) of BGNPY‐R (103.14 μm) and BGNPY‐NR (112.40 μm) and volume mean diameter (*d*_4,3_) of BGNPY‐R (105.46 μm) and BGNPY‐NR (114.86 μm) are illustrated in Table 3. There was no significant (*p* > 0.05) difference in the surface area mean diameter (*d*_3,2_) and volume mean diameter (*d*_4,3_) of BGNPY‐R and BGNPY‐NR. This implies that perceptibly, the particle size and particle size distribution of the BGN yogurt powders were similar.

**Table 3:** Particle size, particle size distribution, and color characteristics of BGNPY‐R and BGNPY‐NR

Powdered yogurt	*d*_3, 2_ (μm)	*d*_4, 3_ (μm)
BGNPY‐R	103.14 ± 17.80^a^	105.46 ± 19.13^a^
BGNPY‐NR	112.40 ± 6.96^a^	114.86 ± 7.75^a^

Note

Mean values ± *SD* of triplicate determinations. Mean values in the same column followed by different letters are significantly (*p* < 0.05) different; BGNPY‐NR: Bambara groundnut powdered yogurt from fresh BGNM; BGNPY‐R: Bambara groundnut powdered yogurt from reconstituted BGNMP; *d*_3, 2_: surface mean diameter; *d*_4, 3_: volume mean diameter.

**Figure 3:**
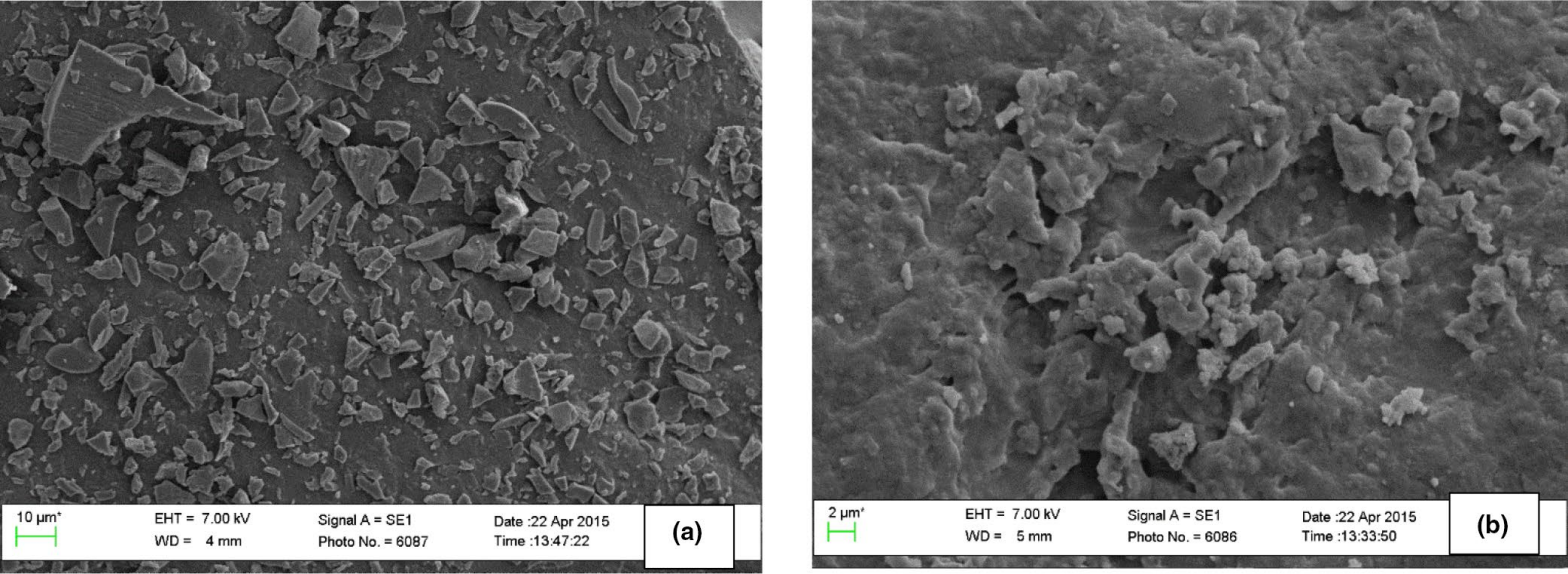
Scanning electron micrographs of Bambara groundnut powdered yogurt—(a) Bambara groundnut powdered yogurt (BGNPY)‐R, (b) BGNPY‐NR

Koç et al. (2014) reported volume mean diameter of a spray dried dairy powder of 3.053 μm, which was quite lower than that of BGNPY‐R (105.46 μm) and BGNPY‐NR (112.40 μm). This difference may be due to different composition and processing technologies. Scanning electron micrographs (SEM) of BGNPY‐R and BGNPY‐NR are shown on Figure 3a. The surface morphology of BGNPY‐R and BGNPY‐NR resembled surface characteristics of a freeze‐dried powder. The surface morphology of the BGN powdered yoghurts differed compared to spray dried powders which are reported to be spherical, with a smooth surface, less collapsed structure, and solid like with the presence of large dents or slightly dimpled surfaces (Fyfe, Kravchuk, Nguyen, Deeth, & Bhandari, 2011).

This may be due to the fact that like freeze‐dried powders, BGNPY‐R and BGNPY‐NR were obtained in a cake or flake like form and required manual grinding into a fine powder. BGNPY‐R (Figure 3a) had a rough surface, with many small cracks, resembling broken glass or flake like appearance. Meanwhile, BGNPY‐NR (Figure 3b) had smoother surface compared to BGNPY‐R. This may be linked to the fact that BGNPY‐R was produced from BGN milk powder diluted with water, which may have resulted in reduced total solids. On the other hand, BGNPY‐NR was produced from fresh BGN milk likely to have higher total solids. In addition, Fyfe et al. (2011) reported that low solid feed results in an increased indentation and collapsed surface structure, whereas high solid feed results in a smoother surface. However, Koç et al. (2014) stated that a porous surface is expected to be advantageous for improved wetting and solubility. The surface structures of BGNPY‐R and BGNPY‐NR were more porous than smooth, thus increasing the potential hydration properties of BGN powdered yoghurts.

#### 3.5.2 Water activity of BGN powdered yogurt

The water activity (*a*_w_) of BGNPY‐R and BGNPY‐NR was 0.46 and 0.40, respectively. The *a*_w_ of the BGNPY‐R and BGNPY‐NR differed significantly (*p* < 0.05) even though the exact same processing was employed to obtain the yogurt powders. Even though the total moisture content value of the BGN powdered yoghurts varied (BGNPY‐R: 9%, BGNPY‐NR: 9.1%), there was no significant difference. Hence, it may not be attributed to the observed water activity variation. However, BGNPY‐R was produced from reconstituted BGN milk powder and BGNPY‐NR was produced from fresh BGN milk. Thus, it was presumed that in the process of reconstitution, various factors such as temperature and vigorous agitation through mixing may be linked to the difference in water activity of the BGNPY yoghurts. Fontana and Campbell (2004) also stated that temperature may cause water activity fluctuation to a food material due to changes in water binding, dissociation of water and sample matrix. Therefore, the significant difference observed may require investigation and monitoring of water activity of the BGN milk prior to inoculation in the future. On the other hand, the *a*_w_ of BGNP‐R and BGNPR‐NR was below the 0.6, as food spoilage due to microorganism is reportedly to rapidly occur at *a*_w_ range 0.6–1.0 (Fontana & Campbell, 2004; Lang et al., 2017). Moreover, the *a*_w_ of the BGNPY‐R (0.46) and BGNPY‐NR (0.40) was comparable to the recommended *a*_w_ range (0.20–0.40) of dried foods (whole milk powder included) making BGN powdered yogurt a potential stable food.

### 3.6 Microbial load characteristics of Bambara groundnut powdered yogurt

The viable starter counts of fermented BGNM‐R and BGNM‐NR before drying were 7.64 and 8.22 log cfu/ml, respectively. Meanwhile, the viable starter counts of foam‐mat dried BGNPY‐R and BGNPY‐NR were observed to be 7.20 and 5.22 log cfu/ml, respectively. BGNPY‐R had a better survival of starter culture, with a reduction percentage of 5.76% while, BGNPY‐NR had a starter culture reduction of 36.50%. BGNPY‐R had much higher microflora survival count in comparison with BGNPY‐NR. This may be explained by varying nutrient compositions of BGNM and BGNMP previously reported by Hardy & Jideani (2017). BGNMP contained a denser nutrient profile (protein: 7.6%, total fat: 1.6%, carbohydrate: 72.4), in comparison with that of BGNM (protein: 0.9%, total fat: 0.3%, carbohydrate: 11.4%). Lactic acid bacteria are facultative anaerobic gram‐positive bacteria reportedly to be bias toward nutrient‐rich food and depend on amino acids and carbohydrates, of which they ferment into organic acids, thus subsequently their rate of multiplication (Lamont, Wilkins, Bywater‐Ekegärd, & Smith, 2017; Sauer, Russmayer, Grabherr, Peterbauer, & Marx, 2017). Cultured foods should supply a daily intake bacterial dosage of 10^8^–10^10^ (8–10 log cfu/ml) in order to be labeled as a probiotic food (Her, Kim, & Lee, 2015), while a minimum dosage of 10^6^ (6 log cfu/ml) may be acceptable (Ng et al., 2011). Thus, BGNPY‐R (7.20 log cfu/ml) can be deemed as a probiotic food, because the number of probiotic bacteria present after drying was within the minimum recommended range, while BGNPY‐NR (5.22 log cfu/ml) cannot be deemed as yogurt. On the other hand, the thermophilic group of lactic acid bacteria are reported to have optimum growth temperature range of 37–40°C, and a maximum temperature of 45–52°C (Husmaini, Abbas, Purwati, Yuniza, & Alimon, 2011). Teixeira (2014) also reported *Lactobacillus bulgaricus species* to have an optimum growth temperature range of 40–50°C and maximum of 62°C. It was thus concluded that the present starter counts observed after drying at 50°C for 24 hr could be thermophilic lactic acid bacteria, known to be heat stable, because of the additional bonds (disulfide linkages, hydrogen bonds, hydrophobic bonds, and ionized group interactions) providing that stability to the secondary and tertiary structure of protein (Kumar & Mishra, 2004a, 2004b).

## 4 CONCLUSION

Bambara groundnut powdered yogurt with considerable quality was produced from reconstituted BGN milk powder, employing the foam‐mat drying processing technology. Even though both the BGN powdered yoghurts (BGNPY‐R and BGNPY‐NR) showed favorable functional rehydration properties (WSI, WAI, wettability), necessary for reconstitution, BGNPY‐R was significantly higher in wettability making it superior in maintaining contact with a liquid. The BGN powdered yoghurts contained a high bulk density, a crucial requirement for commercial food powder, beneficial during packaging and shipping of material. BGNPY‐R and BGNPY‐NR exhibited a fairly satisfactory nutritional profile, revealing to be a source of total dietary fiber and protein. Both the BGN powdered yoghurts had a glass transition temperature well above room temperature (21 ± 2°C) signifying its capability to overcome caking and stickiness. However, after undergoing drying, only BGNPY‐R could be deemed as a probiotic containing food, as the probiotic viability was well above the minimum recommended dosage. It can be concluded that production of BGN powdered yogurt from reconstituted BGN milk powder is feasible.

## CONFLICT OF INTEREST

The authors declare that they have no conflict of interest.

## ETHICAL STATEMENT

In this research work, human participants or animal studies were not necessary. The manuscript is not currently being considered for publication in another journal.
